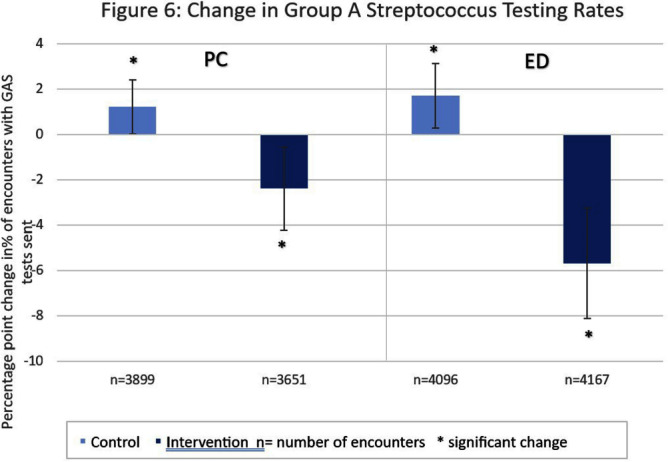# The Impact of Tele-Stewardship on Rural Antibiotic Prescribing Practices

**DOI:** 10.1017/ash.2024.173

**Published:** 2024-09-16

**Authors:** Matthew Peworchik, Ritu Banerjee, Sophie Katz

**Affiliations:** Vanderbilt University; Vanderbilt University Medical Center

## Abstract

**Background:** Antibiotic prescribing for children is highest in rural areas. Tele-stewardship allows for implementation of antimicrobial stewardship (AS) via telecommunication with providers. This study addresses need for better AS in rural areas by implementing and evaluating bundled outpatient AS interventions using tele-stewardship in rural pediatric primary care (PC) clinics and emergency departments (EDs) affiliated with Vanderbilt University Medical Center. **Methods:** The bundle includes (1) patient/guardian educational materials, (2) antibiotic use commitment posters (3) provider education through quarterly teaching pearls and app-based microlearning modules (QuizTime), and (4) quarterly audit/feedback with peer comparison on guideline-concordant antibiotic use via tele-meeting and email. Participants are pediatric prescribers (physician, physician assistant, nurse practitioner). We compared antibiotic prescription data for children < 1 8 years collected during the baseline period (Jan–Dec 2022) to the intervention period (Jan-Sept 2023). Two academic PC clinics and one ED where interventions were not implemented were included as “controls”. The primary outcome is percent of encounters that result in an antibiotic prescription. Secondary outcomes include (1) percent of encounters with guideline-concordant antibiotic choice for otitis media (AOM), streptococcal pharyngitis (GAS), sinusitis, and community-acquired pneumonia (CAP); (2) percent of encounters with 5-day antibiotic duration for AOM, sinusitis, and CAP; and (3) percent of encounters with rapid GAS testing. ED sinusitis data not analyzed due to small N. Significance was determined by calculating 95% confidence intervals for the difference of proportions. **Results:** There were 139,474 PC encounters (91,706 baseline and 47,768 intervention) and 94,205 ED encounters (54,138 baseline and 40,067 intervention) among 20 PC prescribers and 38 ED prescribers from January 2022-September 2023. Compared to baseline, the antibiotic prescription rate decreased 1.1% in intervention PCs but increased 0.9% in control PCs (Figure 1). Compared to baseline, the antibiotic prescription rate decreased by 0.4% in the intervention EDs but increased 3.1% in the control ED (Figure 1). Secondary outcomes showed significantly increased proportions of guideline concordant ED AOM prescriptions, 5-day PC AOM prescriptions (Figure 2), guideline concordant ED streptococcal pharyngitis prescriptions (Figure 3), and guideline concordant PC sinusitis prescriptions (Figure 4). There was a decrease in GAS tests in intervention PCs and EDs (Figure 6). **Conclusions:** Interim analysis shows bundled implementation strategies using tele-AS led to significantly decreased overall antibiotic use in rural PC clinics compared to control sites. The study is ongoing and will continue to evaluate outcomes over a longer intervention period to reduce seasonal bias.

**Disclosure:** Sophie Katz: Research Grant - Pfizer; Research Grant - Dolly Parton Pediatric Infectious Diseases Research Fund; Consultant - Optum

**Figure:**
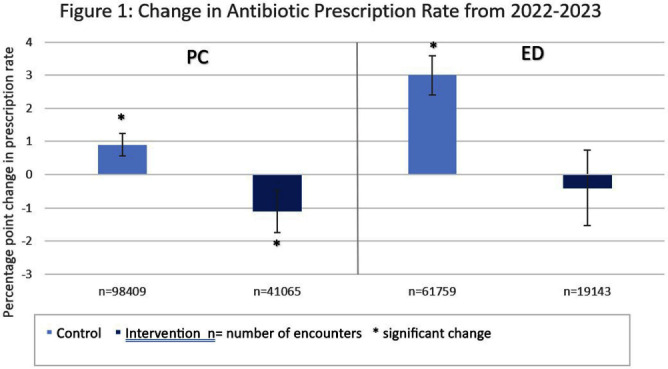

**Figure:**
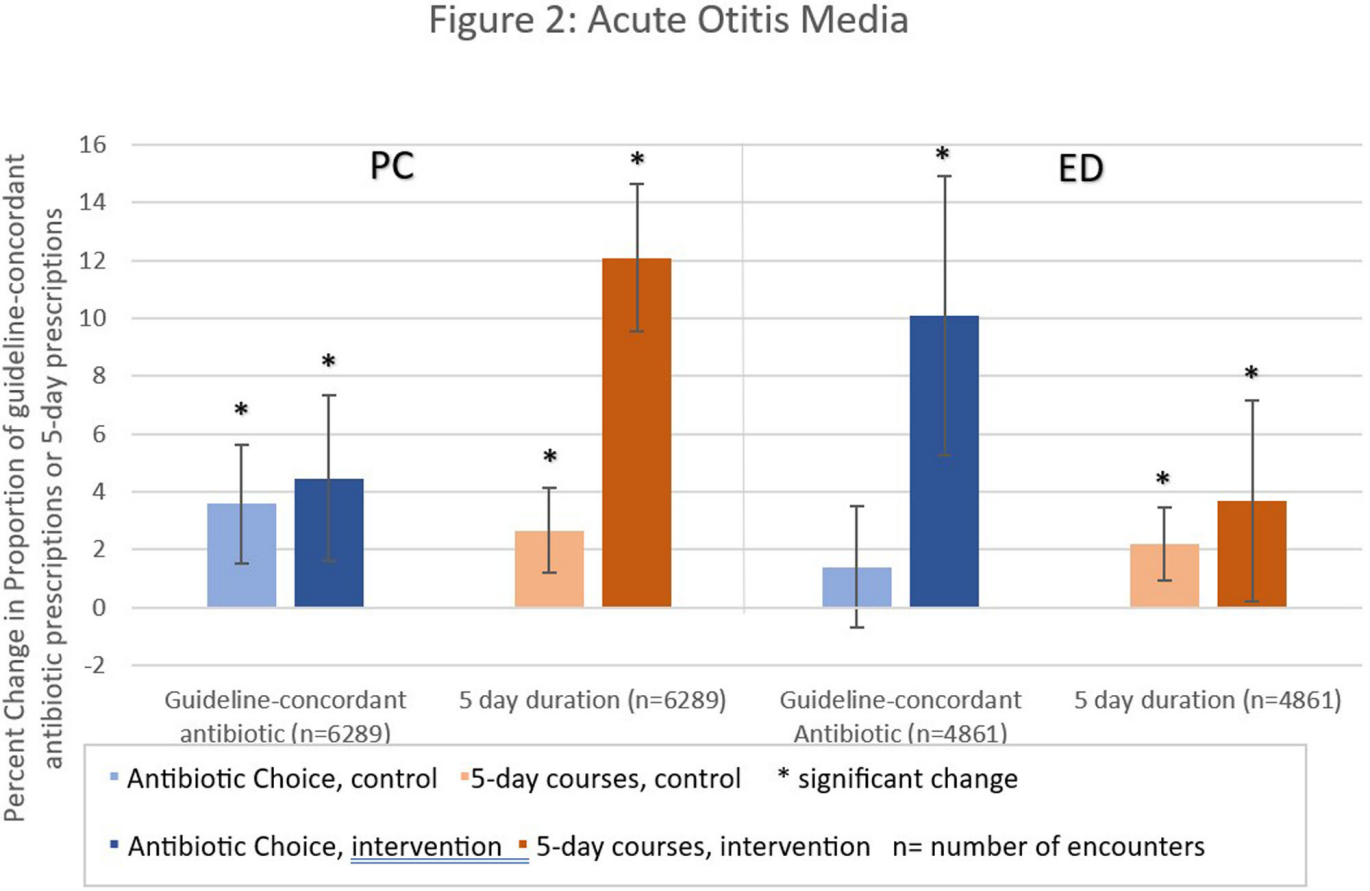

**Figure:**
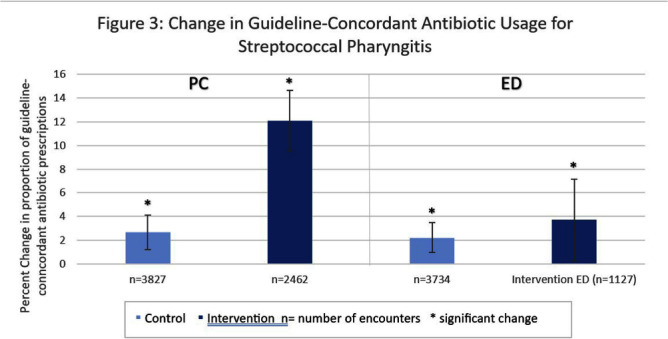

**Figure:**
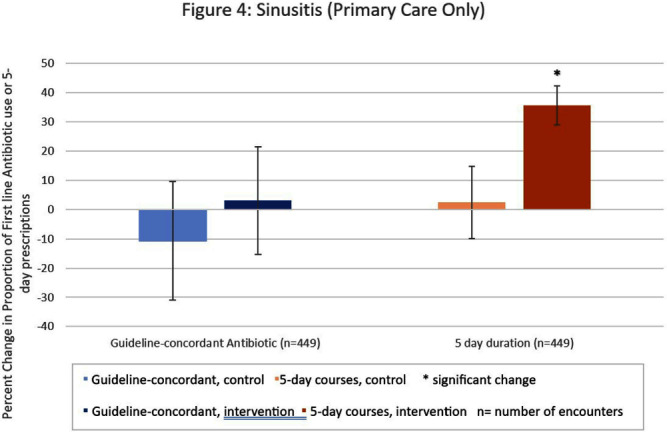

**Figure:**
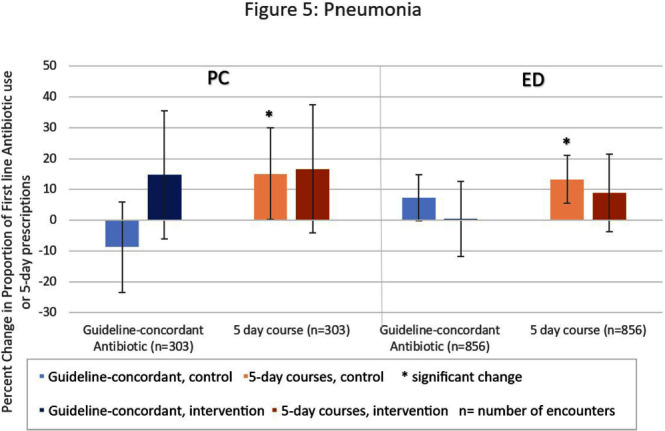

**Figure:**